# Jiao Tai Wan Attenuates Hepatic Lipid Accumulation in Type 2 Diabetes Mellitus

**DOI:** 10.1155/2013/567045

**Published:** 2013-11-10

**Authors:** Zhaoyi Huang, Xiaohu Xu, Fuer Lu, Nan Wang, Guang Chen, Yan Zhao, Xin Zou, Kaifu Wang, Hui Dong, Lijun Xu

**Affiliations:** ^1^Department of Integrated Traditional Chinese and Western Medicine, Tongji Hospital, Tongji Medical College, Huazhong University of Science and Technology, Wuhan, Hubei 430030, China; ^2^Institute of Integrated Traditional Chinese and Western Medicine, Tongji Hospital, Tongji Medical College, Huazhong University of Science and Technology, Wuhan, Hubei 430030, China; ^3^Department of Radiology, Tongji Hospital, Tongji Medical College, Huazhong University of Science and Technology, Wuhan, Hubei 430030, China

## Abstract

Jiao Tai Wan (JTW), a Chinese herbal formula containing Rhizoma Coptidis and Cortex Cinnamomi, has been used for diabetic treatment for many years. The aim of this study was to determine the main components in JTW and to investigate the effects of JTW on hepatic lipid accumulation in diabetic rats and humans. JTW extract was prepared and the main components were assayed by HPLC. An animal model of diabetes mellitus was established and JTW was administered intragastrically. In the clinical study, diabetic patients with poor glycemic control were treated with JTW. Blood glucose and lipid parameters, liver histology, hepatic triglyceride content and lipogenic gene expression were examined. Our data demonstrated that JTW significantly improved hyperglycemia, hyperlipidemia and hepatic lipid accumulation in diabetic rats. This was accompanied by the down-regulation of acetyl coenzyme A carboxylase (ACC) and fatty acid synthase (FAS) protein expressions, and the up-regulation of AMP-activated protein kinase (AMPK) and phosphorylated-ACC (pACC) protein expressions in the liver tissues. Diabetic patients also exhibited decreases in their hepatic triglyceride content. The results suggest that JTW attenuates hepatic lipid accumulation in diabetic rats and humans. These beneficial effects are possibly associated with the inhibition of lipogenic gene expression in the liver.

## 1. Introduction

Obesity, the abnormal or excessive accumulation of fat within the body, has become a global epidemic secondary to high caloric food intake and sedentary life style [1]. Under physiological conditions, excessive free fatty acid (FFA) is converted to triglycerides or cholesterol esters and is then stored in lipid droplets inside adipocytes [2]. The initial deposition of triglycerides occurs in subcutaneous adipose tissue, and as the deposition increases in size, insulin resistance rises and limits further subcutaneous lipid accumulation. Triglycerides are then diverted to the visceral fat depot as well as to nonadipose tissue [3]. The latter is also known as ectopic lipid accumulation and may be linked to insulin resistance and *β* cell dysfunction [4, 5]. In the skeletal muscle and liver, fat deposition has been associated with the development of insulin resistance [6, 7]. In the pancreatic islets, FFA and triglycerides accumulation induce *β* cell functional impairment and lipoapoptosis [8]. Therefore, ectopic lipid accumulation is often associated with the development of type 2 diabetes mellitus (T2DM).

Excessive lipid accumulation in the liver of patients who drink little or no alcohol is called nonalcoholic fatty liver disease (NAFLD). NAFLD refers to a wide spectrum of liver diseases ranging from simple hepatic steatosis to nonalcoholic steatohepatitis to cirrhosis. NAFLD is highly prevalent in subjects with T2DM; approximately 70% of type 2 diabetic patients are estimated to have NAFLD [9]. This high prevalence rate makes the treatment of diabetes complex due to the liver damage and hepatotoxicity of oral hypoglycemic drugs. To date, there has been no specific or effective treatment of NAFLD, especially in the patients with T2DM. A few clinical trials of drugs designed to reduce insulin resistance, including thiazolidinediones and metformin, appear to have some limited benefits [10, 11]. However, the beneficial effects of insulin sensitizers should be further confirmed by more multicenter randomized clinical trials. 

Jiao Tai Wan (JTW) is a classical traditional Chinese prescription first mentioned in the book Han-Shi-Yi-Tong in the Ming Dynasty that consists of Rhizoma Coptidis (*Coptis chinensis *Franch, Ranunculaceae) and Cortex Cinnamomi(*Cinnamomum cassia* Presl, Lauraceae). Rhizoma Coptidis (huang lian in Chinese) mainly consists of various alkaloids including berberine, coptisine, palmatine, and epiberberine, whereas Cortex Cinnamomi (rou gui in Chinese) largely contains cinnamaldehyde and cinnamic acid [12, 13]. JTW is a common remedy to treat insomnia caused by the imbalance between the heart and kidney. However, recent research has determined that Rhizoma Coptidis and Cortex Cinnamomi both exert beneficial effects in diabetes [14–18]. We have also demonstrated that JTW is more effective than its single components in the treatment of rat hyperglycemia [19]. Given that fatty liver is highly prevalent in diabetes, we hypothesize that JTW may have the potential to alleviate hepatic fat accumulation in diabetes. Therefore, we investigated the effect of JTW on hepatic fat content in diabetic rats and humans.

## 2. Materials and Methods

### 2.1. Animals and Experimental Design

Male Wistar rats (8 weeks, 180 ± 20 g) were obtained from the Hubei Province Center for Disease Control and Prevention (Wuhan, China). The rats received a standard diet with free access to tap water and were maintained on a 12:12 h light-dark cycles in a humidity- and temperature-controlled environment. All procedures were performed in accordance with the Chinese guidelines for animal experiments (MSTPRC Directive of 1988, no. 88-2) and were approved by the local authorities.

Seven rats were selected randomly as the control group and were injected with saline into the tail vein. The remaining rats were injected with streptozotocin (STZ; Sigma Chemical Co. MO, USA; 30 mg/kg) into the tail vein. An oral glucose tolerance test (OGTT) was performed two weeks later. Impaired glucose tolerance (IGT) was diagnosed when the plasma glucose levels of rats at two time points were higher than the upper limit (4.62 mmol/L before glucose-loading, 7.56 mmol/L at 1 h after glucose-loading, and 6.29 mmol/L at 2 h after glucose-loading, resp., which is the 95% range of confidence calculated according to plasma glucose levels in the rats of control group) or when the plasma glucose levels at one time point exceeded the upper limit by more than 20% (5.54 mmol/L, 9.07 mmol/L, and 7.55 mmol/L, resp.). Thereafter, twenty-one rats with IGT were randomly divided into 3 groups. Rats receiving saline served as diabetic controls. Rats receiving JTW (3 g/kg/day) were taken as the JTW group, and rats receiving metformin hydrochloride (183 mg/kg/day) were taken as the metformin (Met) group. Oral gavage was performed once a day between 8:00 and 10:00 a.m. The doses were adjusted to the body weight, which was recorded once a week. Meanwhile, the high-fat chow (containing 72.7% standard laboratory rat chow, 20% lard, 5% egg yolk powder, 2% cholesterol, and 0.3% bile salts) was provided to the animals with IGT while the rats in the control group continued their standard diet. At the end of the 8-week period, overnight-fasted rats were weighed and then anaesthetized by pentobarbital sodium. Blood samples were obtained from the abdominal aorta. The liver was quickly excised and divided into two parts. One sample was immediately stored in liquid nitrogen, while the other was fixed with 4% paraformaldehyde. 

### 2.2. Preparation of JTW

Rhizoma Coptidis and Cortex Cinnamomi used for animal experiments were obtained from the Traditional Chinese Medicine Company in Hubei Province (Wuhan, China). The voucher specimen was deposited at the herbarium of the Pharmacy Faculty at the Hubei University of Chinese Medicine and authenticated by Department of Pharmacognosy, Hubei University of Chinese Medicine (Wuhan, China). The rat doses of Rhizoma Coptidis and Cortex Cinnamomi were obtained by the conversion of the human doses (Chinese Pharmacopoeia, 2010) to rat equivalent doses based on body surface areas. The JTW preparation process was as follows: ninety grams Rhizoma Coptidis and forty-five grams Cortex Cinnamomi were mixed together. The mixture was decocted twice by refluxing it with water (1 : 10 w/v), 2 hours for the first time and 1 hour for the second time. The volatile oil was collected by the volatile oil extractor during the decocting procedure. The volatile oil and the solution obtained were concentrated to 450 grams. This extract represented the solution for rat administration and was stored at 4°C until use.

In the clinical study, we used another form of JTW for the convenience of oral administration. JTW, containing Rhizoma Coptidisand Cortex Cinnamomi concentrated granules, was purchased from China Resources Sanjiu Medical and Pharmaceutical Co., Ltd. Rhizoma Coptidis concentrated granules largely contain berberine (>104.0 mg/g, HPLC), coptisine (>26.0 mg/g, HPLC), palmatine (>26.0 mg/g, HPLC), and epiberberine (>17.0 mg/g, HPLC). Cortex Cinnamomi is mainly composed of cinnamaldehyde (>1.0 mg/g, HPLC) and cinnamic acid (>0.5 mg/g, HPLC). Each pack of JTW contains two grams of Rhizoma Coptidis concentrated granules (equal to six grams of Rhizoma Coptidis) and one gram of Cortex Cinnamomi concentrated granules (equal to three grams ofCortex Cinnamomi). Patients mixed the granules together by boiling water and drank two packs each day.

### 2.3. Determinations of the Contents of Berberine, Cinnamaldehyde, and Cinnamic Acid Contents in JTW

Berberine, cinnamaldehyde, and cinnamic acid, the main components in JTW extracts and concentrated granules, were all measured using the high-performance liquid chromatography (HPLC) method. HPLC (Waters, USA) equipped with Waters 600 Pump, and Waters 2487 Dual *λ* Absorbance Detector, and a XTerra RP18 chromatographic column (250 mm × 4.6 mm, 5 *μ*m particle size) was used. The extract of JTW was dissolved in methanol. To measure berberine, all analyses were performed at a column temperature of 35°C. The mobile phase was 30 : 70 (v/v) acetonitrile-water containing potassium dihydrogen phosphate (0.1 mol/L) and sodium hydroxide (0.05 mol/L), adjusted to pH 9.5 with triethanolamine. The flow rate was 0.9 mL/min and the wavelength of detection was set at 345 nm. To measure cinnamaldehyde, the analyses were performed at a column temperature of 22°C. The mobile phase was 55 : 15 : 25 : 5 (v/v/v/v) water, acetonitrile, methanol, and tetrahydrofuran. The flow rate was 0.6 mL/min and the wavelength of detection was set at 290 nm. To measure cinnamic acid, the analyses were performed at a column temperature of 25°C. The mobile phase was 65 : 15 : 15 : 5 (v/v/v/v) water, acetonitrile, methanol, and tetrahydrofuran, adjusted to pH 5.5 with glacial acetic acid. The flow rate was 0.9 mL/min and the wavelength of detection was set at 270 nm. 

### 2.4. Oral Glucose Tolerance Test (OGTT)

The rats were fasted overnight and given a single dose of 50% glucose solution one week before being sacrificed. The glucose solution was administered by gavage at a dose of 2.0 g/kg body weight. Blood samples for glucose measurement were obtained from the tail vein at 0 hour (before glucose-loading), 1 hour, and 2 hours (after glucose-loading) by glucose-oxidase method using a glucose monitor (LifeScan Inc., J&J Company, Milpitas, CA, USA). 

### 2.5. Biochemical Analysis

The plasma levels of total cholesterol (TC), triglycerides (TG), low-density lipoprotein cholesterol (LDL-C), high-density lipoprotein cholesterol (HDL-C), nonesterified fatty acid (NEFA), alanine aminotransferase (ALT), and aspartate aminotransferase (AST) were determined using commercial reagents (Jiancheng Bioengineering Institute, Nanjing, China). The fasting insulin concentration (FINS) was measured by radioimmunoassay kit (Northern Institute of Biotechnology, Beijing, China). The homeostasis model assessment index (HOMA-IR) was calculated using the formula of fasting glucose (mmol/L) × fasting insulin (*μ*IU/mL)/22.5. 

### 2.6. Liver Histology and Hepatic Triglyceride Content Measurements

Serial 4 *μ*m thick paraffin sections of the liver were stained with hematoxylin-eosin (HE) for liver pathology. The morphology of the liver cells and inflammatory cells was observed under high-power magnification (×400). Neutral lipids were extracted from each aliquot using the Folch procedure with slight modifications [20]. Hepatic TG content (HTC) was measured with the same enzymatic kit used in the plasma analysis. 

### 2.7. Western Blot Analysis

Protein lysates extracted from liver homogenates were subjected to 10% SDS-PAGE gel (100 v, 2 h–2.5 h) and then transferred to nitrocellulose membranes. The membranes were blocked with 5% (w/v) nonfat milk in tris-buffered saline with Tween 20 (TBST) for 2 h at room temperature. The blocking solution was then removed, and the membranes were washed by TBST, followed by overnight incubation at 4°C with the primary antibodies (rabbit anti-rat AMPK, pAMPK, ACC, pACC, and FAS) (Abcam, Hong Kong, China) and *β*-actin (Gene Tex Inc., CA, USA). The membranes were then washed by TBST, followed by lucifugal incubation with the DyLight 800-labeled antibody to rabbit IgG (1 : 1000) (KPL Company, Hong Kong, China) at room temperature for 1 h. The membranes were washed in a lucifugal way 3 times with TBST. Protein bands were detected by the LI-COR Odyssey Infrared Fluorescent Scanner (Odyssey, Lincoln, USA). Band densities were determined by Bio-Rad Quantity One software v 4.62 and quantified as the ratio between the OD value of target band and the OD value of *β*-actin.

### 2.8. Clinical Intervention Study

Type 2 diabetic patients with poor glycemic control were enrolled in this study based on the following inclusion criteria: (1) aged 25–70 years; (2) use of stabilizing oral hypoglycemic drugs or lifestyle modification to control their blood glucose for at least 8 weeks; (3) fasting plasma glucose (FPG) 7.0–13.0 mmol or 2-hour postprandial blood glucose (2hPPG) 11.1–16.6 mmol/L and hemoglobin A1c (HbA1c) >6.5%; (4) no known acute or chronic disease based on history, physical examination, and standard laboratory tests; (5) alcohol consumption <20 g/day; and (6) no evidence of hepatitis A, B, or C as well as no evidence of autoimmune hepatitis, clinical signs or symptoms of inborn errors of metabolism, and no history of the use of toxins or drugs known to induce hepatitis. Exclusion criteria included thyroid disease, the use of antihypertensive agents that could possibly influence glucose metabolism (*β*-blockers and thiazides), the use of insulin, and women who were pregnant or lactating. The nature and potential risks of the study were explained to all subjects before obtaining their written informed consent. The experimental protocol was approved by the ethics committee of the Tongji Hospital affiliated with Tongji Medical College following the ethical principles outlined in the Declaration of Helsinki.

A total of forty patients with T2DM were enrolled. None of patients withdrew or dropped out of the study during the 12 weeks. The patients were randomly divided into two groups: JTW group (treated with one pack of JTW two times a day for 12 weeks) and the control group. Their previous diabetes management was maintained during the 12-week experimental period except for the addition of JTW in patients of the JTW-treated group. The following indicators before and after treatment were measured: body mass index (BMI), waist circumference, waist-to-hip ratio (WHR), blood pressure (BP), FPG, 2hPPG, HbA1c, plasma TC, TG, LDL-C, HDL-C, ALT, and AST. 

All of the patients received proton magnetic resonance spectroscopy (^1^H-MRS) scans of their livers before and after treatment, as described previously [21], which was acquired on a 1.5-T MRI scanner (GE Healthcare, USA) using a standard body coil and respiratory gating mode in our hospital. Spectroscopic data were processed using the SAGE software (GE Healthcare, USA). Chemical shifts were measured relative to water at 4.77 ppm. The methylene signal, which represents intracellular triglyceride, was measured at 1.40 ppm. The peak areas of the water (Sw) and fat resonance (Sf) were measured. HTC was calculated by dividing 100 times Sf by the sum of Sf and Sw. All spectra were analyzed by physicists who were unaware of any of the clinical data.

### 2.9. Statistical Analysis

Normally distributed data are presented as the mean ± SD, whereas nonnormally distributed data are presented as the median with the 25th and 75th percentiles in parenthesis. Statistical significance was determined by one-way analysis of variance (ANOVA) followed by Dunnett's T3 test for data with equal variances not assumed. For data with equal variances assumed, ANOVA followed by LSD test was used. Nonparametric tests were used to compare median values between the groups. Calculations were performed using SPSS 14.0 software. Statistical significance was defined as *P* less than 0.05.

## 3. Results

### 3.1. The Content of Berberine, Cinnamaldehyde, and Cinnamic Acid in JTW

**Figure 1:**
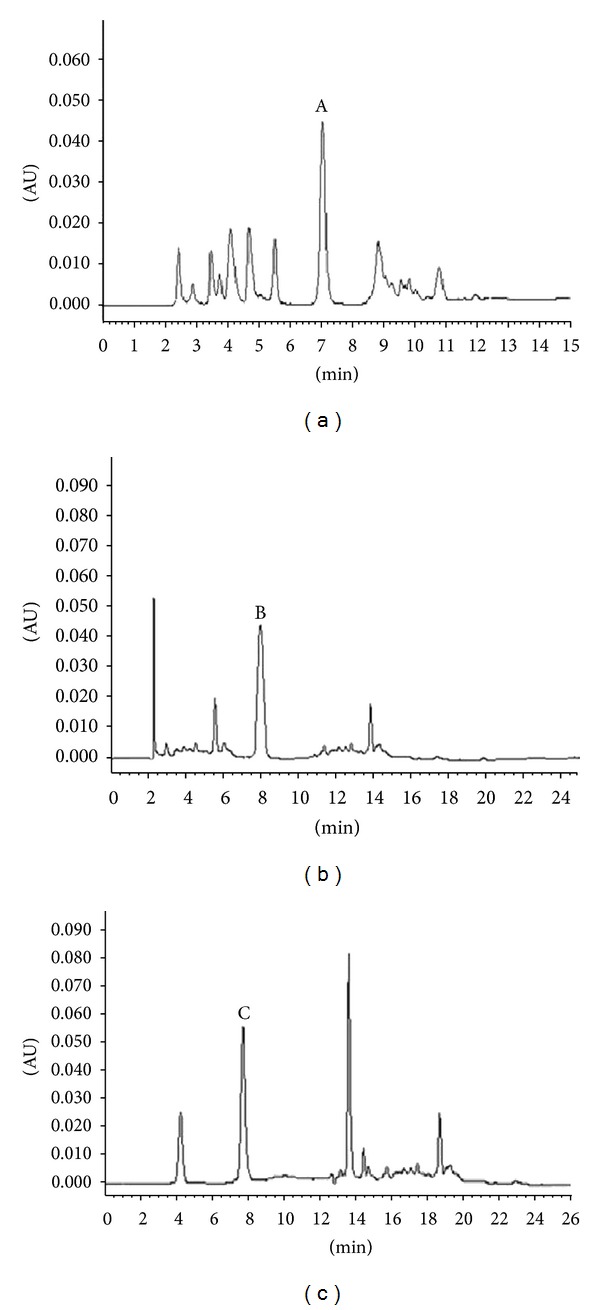
The chromatograms of berberine, cinnamaldehyde, and cinnamic acid in JTW. (a) The chromatogram of berberine in JTW (peak A is berberine), (b) the chromatogram of cinnamaldehyde in JTW (peak B is cinnamaldehyde), and (c) the chromatogram of cinnamic acid in JTW (peak C is cinnamic acid).

The contents of berberine, cinnamaldehyde, and cinnamic acid in the JTW extract used in the animal experiments were 23.03 mg/g, 3.12 mg/g, and 0.27 mg/g, respectively. In clinical trials, the contents of berberine, cinnamaldehyde, and cinnamic acid in the JTW concentrated granules were 79.51 mg/g, 13.39 mg/g, and 1.06 mg/g, respectively. The chromatograms of berberine, cinnamaldehyde, and cinnamic acid in JTW were presented in Figure 1.

### 3.2. The Effect of JTW on Body Weight, OGTT, Insulin Levels, and HOMA-IR in Diabetic Rats

As shown in Table 1, in diabetic rats, body weight, fasting and postprandial plasma glucose (FPG, 2hPPG), FINS, and HOMA-IR were all increased compared with the respective levels in the control group (*P* < 0.05, *P* < 0.01). In contrast to diabetic rats, the rats in the JTW- or metformin-treated group exhibited little gain in body weight as well as a significant reduction in plasma glucose, insulin levels, and HOMA-IR (*P* < 0.05, *P* < 0.01).

### 3.3. The Effect of JTW on Plasma Lipid Profiles in Diabetic Rats

As shown in Table 2, diabetic rats exhibited severe dyslipidemia characterized by elevated TC, TG, LDL-C, and NEFA levels as well as decreased HDL-C levels compared with control group (*P* < 0.01). However, the treatment with JTW or metformin significantly reversed these plasma lipid changes in diabetic rats (*P* < 0.05, *P* < 0.01).

### 3.4. The Effect of JTW on Plasma Liver Enzymatic Activity, HTC, and Liver Histology in Diabetic Rats

**Figure 2:**
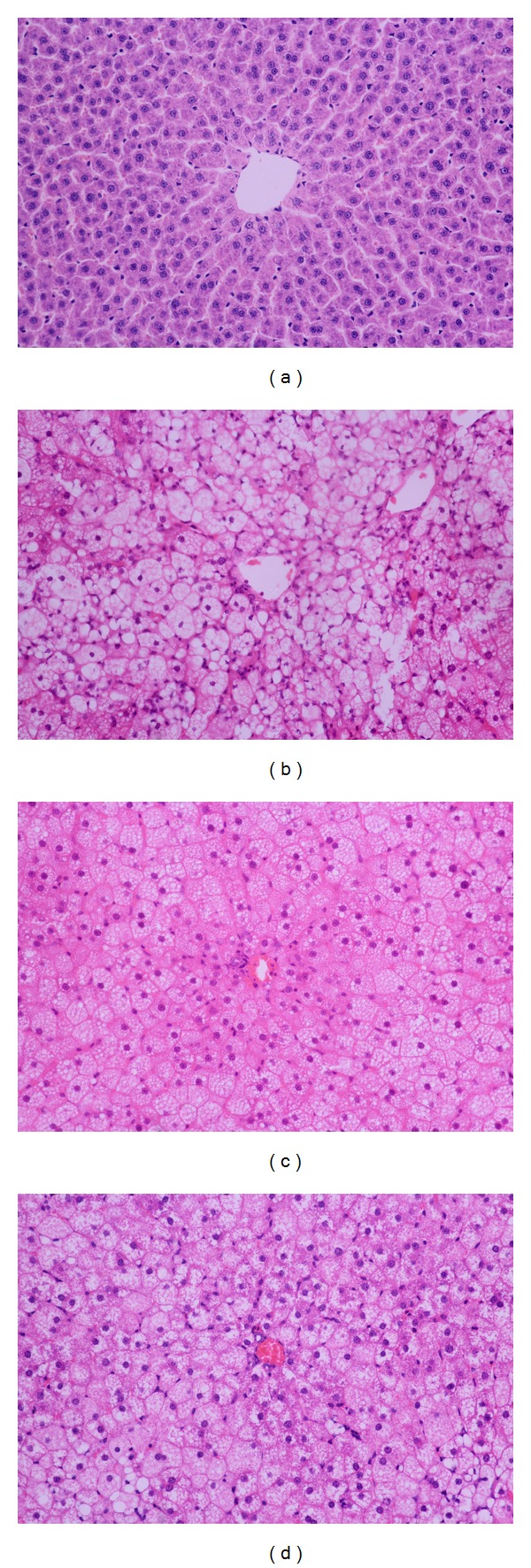
The effect of JTW on liver pathology in diabetic rats. Representative H&E stained images in liver sections from the control group (a), diabetic group (b), JTW group (c), and metformin group (d). Original magnification ×400.

As shown in Table 3, diabetic rats exhibited a larger increase in plasma ALT, AST, and HTC (*P* < 0.01). These changes were accompanied by a larger increase in the severity of hepatic steatosis compared with controls as well as increased intrahepatic inflammatory cell infiltration (Figures 2(a) and 2(b)). However, JTW or metformin treatment significantly reduced ALT, AST, and HTC (*P* < 0.05, *P* < 0.01). The degree of fatty degeneration was decreased without obvious intrahepatic inflammatory cell infiltration (Figures 2(c) and 2(d)). 

### 3.5. The Effect of JTW on the Liver Expression of Genes Related to Lipogenesis in Diabetic Rats

**Figure 3:**
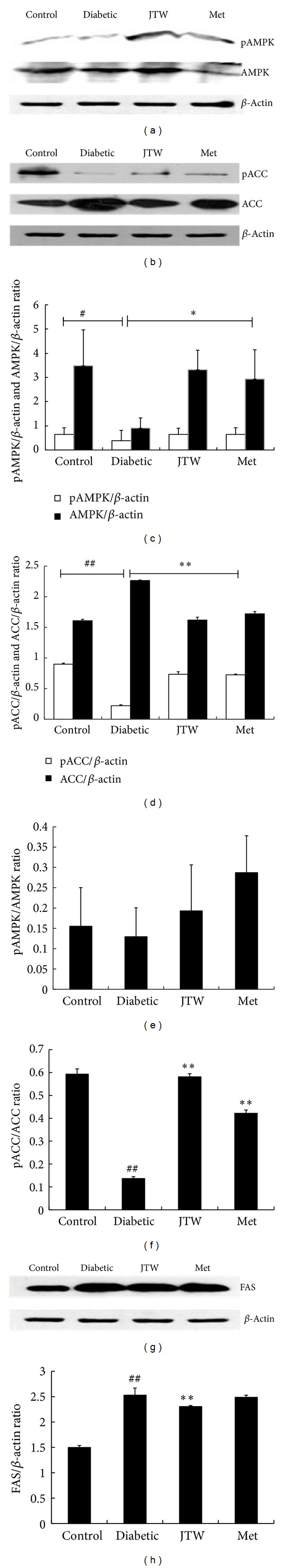
The effect of JTW on hepatic lipogenic gene expression in diabetic rats. pAMPK and AMPK protein expressions ((a), (c), and (e)), pACC and ACC protein expressions ((b), (d), and (f)), and FAS protein expression ((g), (h)). Values are expressed as mean ± SD. ^#^ *P* < 0.05 and ^##^ *P* < 0.01 versus control group. *P < 0.05 and **P < 0.01 versus diabetic group.

As shown in Figure 3, the hepatic expression of AMPK, pAMPK, and pACC in diabetic rats was decreased compared with controls. This decrease was accompanied by increased hepatic expression of ACC and FAS (*P* < 0.05, *P* < 0.01). However, treatment with JTW or metformin resulted in a marked increase in the hepatic expression of AMPK, pAMPK, and pACC as well as significant decreases in ACC and FAS proteins (*P* < 0.05, *P* < 0.01). JTW treatment did not change the ratio of pAMPK to AMPK expression but increased the ratio of pACC to ACC expression significantly (*P* < 0.01).

### 3.6. The Effect of JTW on Clinical Markers and HTC in Diabetic Patients with Poor Glycemic Control

No significant differences were noted between the baseline markers of diabetic patients in the JTW group and the control group. Patients with poor glycemic control were treated with JTW for 12 weeks. These patients exhibited decreasing trend in their BMI, waist circumference, WHR, FPG, 2hPPG, FINS, HbA1c, TC, TG, and LDL-C at the end of the treatment period (*P* < 0.05). In addition, HTC decreased significantly after the addition of JTW to the diabetic treatment but increased in the control group (*P* < 0.05). However, the levels of ALT, AST, and HDL-C did not change significantly (Table 4).

### 3.7. Adverse Effects

No serious adverse events were observed when JTW was used. Plasma creatinine concentrations and electrocardiography (ECG) were also monitored in the clinical study. No significant changes in plasma creatinine were observed during the 12 weeks of JTW treatment. No severe hypoglycemia was observed.

## 4. Discussion

Chinese medicine is an excellent alternative and complementary medicine in treating NAFLD. In traditional Chinese medicine, NAFLD falls into the category of symptoms caused by phlegm/dampness retention, qi/blood stasis, and spleen/kidney deficiency. Increasing evidence indicates that herbs that remove accumulated pathogenic phlegm and dampness are useful in treating NAFLD [22]. In Chinese medicine, Rhizoma Coptidis has the potential to remove dampness and Cortex Cinnamomican reinforce kidney deficiency, both of which are involved in the development of NAFLD. Additionally, the hypoglycemic and hypolipidemic effects of Rhizoma Coptidis and Cortex Cinnamomi have been confirmed by clinical practice and experimental studies [15, 23, 24]. Among the main constituents in JTW extract, berberine, cinnamaldehyde, and cinnamic acid have also been demonstrated to exhibit beneficial effects in attenuating disturbances of glucose and lipid metabolism [17, 25, 26]. However, the effect of JTW, which is the combination of these two herbs, on treating NAFLD has not been sufficiently reported. Thus, the present study aimed to explore the effect of JTW on hepatic lipid accumulation in rats and humans with T2DM.

In the animal study, the diabetic rats exhibited the characteristics of hyperglycemia, hyperinsulinemia, and hyperlipidemia. Plasma liver enzymatic activity and HTC also significantly increased. Liver histology demonstrated severe fatty degeneration and inflammation infiltration. The oral administration of JTW not only attenuated the biochemical disturbances in peripheral blood but also alleviated TG elevation and the morphological changes in liver tissues. We suggest that JTW could be an effective herbal prescription to treat nonalcoholic steatohepatitis of diabetic rats. These results were further confirmed by the clinical study, with the exception of the effects of JTW on liver histology and enzymatic activity. Invasive liver biopsy was not used in the clinical study to obtain histology data for ethical considerations. As an alternative, we applied noninvasive ^1^H-MRS scans of liver as an assessment tool. Previous studies have demonstrated that, although this method gives no information of liver histology, HTC obtained by spectroscopy closely coincides with biopsy or autopsy-derived triglyceride concentrations [27]. Additionally, ALT levels do not correlate well with HTC of NAFLD severity in many reports. Patients with NAFLD and normal ALT values may also exhibit steatohepatitis and advanced fibrosis [28]. However, the preliminary data from our clinical study suggests that, at minimum, JTW has beneficial effects on blood glucose and lipid control and also exhibits efficacy in reducing hepatic lipid accumulation in diabetic patients. 

With respect to the constituents of Rhizoma Coptidis and Cortex Cinnamomi, many substances have been identified. Among the constituents effective in treating diabetes, berberine (which is contained in Rhizoma Coptidis), cinnamaldehyde, and cinnamic acid (which are contained in Cortex Cinnamomi) have attracted significant attention [13, 29, 30]. Unlike berberine and cinnamic acid, cinnamaldehyde is difficult to detect in peripheral blood after taking cinnamon. This is likely due to the rapid oxidation of cinnamaldehyde to cinnamic acid by oxidase after absorption [31]. To date, there has been no direct clinical evidence supporting the efficacy of Rhizoma Coptidis and Cortex Cinnamomi or the above-mentioned constituents in reducing HTC. However, the potential efficacy of Cortex Cinnamomi extract and berberine has been identified in several experimental studies [32–34]. Berberine moderates glucose and lipid metabolism through a multipathway mechanism [35]. One of the possible mechanisms may be related to the activation of hepatic adenosine monophosphate-activated protein kinase (AMPK). AMPK is a serine/threonine protein kinase that plays a central role in regulating cellular metabolism and energy balance [36]. Berberine, through AMPK activation, inhibits triglyceride synthesis and increases fatty acid oxidation by modulating downstream-signaling component expression and activity, leading to the alleviation of hepatosteatosis [37, 38]. In the present study, hepatic AMPK, pAMPK, and pACC protein expression were all upregulated in rats treated with JTW. This was accompanied by the down-regulation of ACC and FAS expressions. However, there was no change in the ratio of pAMPK to AMPK expression although the ratio of pACC to ACC expression increased significantly. These data suggest that JTW could inhibit lipogenesis in liver tissues. Thus hepatic TG content decreased after JTW intervention. 

However, one limitation of our study must be considered. The effect of JTW on the fatty liver was evaluated using different formulations in the animal and clinical studies. In the animal experiments, the traditional Chinese herbal decoction was used, whereas concentrated herbal granules were chosen in the clinical trial. These different preparations account for the differences in the content of berberine, cinnamaldehyde, and cinnamic acid in JTW detected by HPLC. However, the daily dosage of JTW intake in diabetic rats was nearly identical to that in diabetic patients. Herbal granules, extracted from herbs with modern pharmaceutical technology, are convenient for those who cannot make an herbal decoction every day. We believe that the application of JTW granules assures quality control in the clinical trials. Nonetheless, we cannot confirm that there are any other differences in the constituents of JTW without the process of boiling. However, we have provided preliminary data that suggest that JTW is promising in the treatment of NAFLD. 

In summary, our study demonstrates that oral treatment with JTW attenuates hepatic lipid accumulation in diabetic rats and humans. These beneficial effects are possibly associated with the inhibition of lipogenic gene expression in the liver.

## Figures and Tables

**Table 1:** The effect of JTW on body weight, OGTT, insulin levels, and HOMA-IR in diabetic rats.

Group	Body weight	FPG	1hPPG	2hPPG	FINS	HOMA-IR
	(g)	(mmol/L)	(mmol/L)	(mmol/L)	(*μ*IU/mL)	
Control	451 ± 28	6.20 ± 0.69	7.39 ± 0.50	7.10 ± 0.44	29.71 ± 10.21	9.10 ± 1.37
Diabetic	509 ± 52^#^	8.34 ± 0.81^##^	13.39 ± 4.80^##^	11.63 ± 1.93^##^	55.32 ± 19.18^##^	17.54 ± 4.37^##^
JTW	459 ± 40*	6.13 ± 0.54**	8.71 ± 0.78**	8.03 ± 0.82**	40.38 ± 11.86**	11.14 ± 1.93**
Met	435 ± 49*	6.30 ± 0.69**	8.17 ± 1.45**	7.76 ± 1.15**	41.15 ± 11.86**	11.47 ± 2.83**

Values are expressed as mean ± SD. ^#^ *P* < 0.05 and ^##^ *P* < 0.01 versus control group. *P < 0.05 and **P < 0.01 versus diabetic group.

**Table 2:** The effect of JTW on plasma lipid profiles in diabetic rats.

Group	TC	TG	LDL-C	HDL-C	NEFA
	(mmol/L)	(mmol/L)	(mmol/L)	(mmol/L)	(mmol/L)
Control	3.16 ± 0.18	0.34 ± 0.03	1.20 ± 0.18	1.12 ± 0.09	0.81 ± 0.28
Diabetic	5.66 ± 0.77^##^	0.95 ± 0.28^##^	3.76 ± 1.12^##^	0.83 ± 0.13^##^	1.57 ± 0.35^##^
JTW	4.23 ± 0.34**	0.45 ± 0.03**	1.92 ± 0.45*	1.09 ± 0.21**	1.15 ± 0.30*
Met	3.88 ± 0.62**	0.45 ± 0.04**	1.58 ± 0.70**	1.07 ± 0.18**	1.02 ± 0.41**

Values are expressed as mean ± SD. ^##^ *P* < 0.01 versus control group. *P < 0.05 and **P < 0.01 versus diabetic group.

**Table 3:** The effect of JTW on plasma liver enzyme activity and HTC in diabetic rats.

Group	ALT	AST	HTC
	(U/L)	(U/L)	(*μ*mol/g)
Control	36.13 ± 0.39	18.13 ± 4.36	4.16 ± 2.54
Diabetic	58.62 ± 10.45^##^	29.62 ± 3.67^##^	12.30 ± 3.30^##^
JTW	41.43 ± 2.08**	21.98 ± 3.62**	8.35 ± 3.20*
Met	42.55 ± 3.17*	22.07 ± 3.19*	8.70 ± 3.22*

Values are expressed as mean ± SD. ^##^ *P* < 0.01 versus control group. *P < 0.05 and **P < 0.01 versus diabetic group.

**Table 4:** General characteristics and laboratory data of diabetic patients at baseline and 12 weeks after the therapy.

Group	Control		JTW	
	Before	After	Before	After
Subjects (M/F)	9/11		9/11	
Age (years)	46.1 ± 7.3		46.2 ± 6.1	
BMI (kg/m^2^)	24.9 ± 3.5	24.7 ± 3.3	25.1 ± 3.6	22.9 ± 2.9^#∗^
WC (cm) (F)	83.4 ± 5.7	83.3 ± 6.5	82.9 ± 5.8	80.6 ± 4.2^#∗^
WC (cm) (M)	90.6 ± 7.0	90.5 ± 8.1	91.7 ± 6.7	89.1 ± 5.6^#∗^
WHR (F)	0.87 ± 0.06	0.87 ± 0.05	0.88 ± 0.05	0.86 ± 0.03^#∗^
WHR (M)	0.94 ± 0.07	0.93 ± 0.07	0.94 ± 0.04	0.91 ± 0.05^#∗^
SBP (mmHg)	132 ± 22	130 ± 24	130 ± 20	130 ± 20
DBP (mmHg)	80 ± 12	79 ± 10	80 ± 10	79 ± 12
FPG (mmol/L)	7.64 (6.20, 9.37)	7.67 (6.41, 9.25)	7.89 (6.10, 9.22)	6.78 (4.01, 8.69)^#∗^
2hPPG (mmol/L)	13.16 ± 4.15	13.21 ± 4.55	13.25 ± 4.09	10.55 ± 3.51^#∗^
FINS (*μ*IU/L)	7.59 (4.33, 10.42)	7.61 (4.57, 9.99)	7.52 (4.11, 12.92)	6.04 (4.20, 9.61)^#∗^
HbA1c (%)	7.81 ± 1.61	7.80 ± 1.59	7.84 ± 1.33	6.91 ± 1.48^#∗^
TC (mmol/L)	4.76 ± 0.73	4.82 ± 0.88	4.94 ± 0.83	3.89 ± 0.63^#∗^
TG (mmol/L)	1.32 (1.05, 2.38)	1.31 (1.01, 2.23)	1.29 (1.02, 2.75)	1.18 (0.94, 2.13)^#∗^
HDL-C (mmol/L)	1.24 (0.98, 1.58)	1.25 (1.03, 1.60)	1.27 (1.03, 1.90)	1.26 (0.96, 1.87)
LDL-C (mmol/L)	2.89 ± 0.82	2.90 ± 0.74	2.95 ± 0.76	1.87 ± 0.77^#∗^
ALT (U/L)	26 (18, 35)	25 (17, 35)	21 (18, 40)	24 (17, 35)
AST (U/L)	22 (15, 31)	21 (16, 32)	19 (11, 35)	21 (10, 30)
HTC (%)	9.63 (5.87, 11.18)	10.79 (6.75, 19.99)^#^	9.81 (5.76, 15.09)	6.54 (4.82, 13.08)^#∗^

Normally distributed data are presented as the mean ± SD; nonnormally distributed data are presented as the median with the 25th and 75th percentiles in parenthesis. ^#^ *P* < 0.05 versus pretreatment. *P < 0.05 versus the control group.

## Abbreviations

ACC: Acetyl coenzyme A carboxylase
ALT: Alanine aminotransferase
AMPK: AMP-activated protein kinase
BMI: Body mass index
DBP: Diastolic blood pressure
FAS: Fatty acid synthase
FFA: Free fatty acid
FINS: Fasting insulin level
FPG: Fasting plasma glucose
HbA1c: Hemoglobin A1c
HDL-C: High-density lipoprotein cholesterol
^1^H-MRS: Proton magnetic resonance spectroscopy
HOMA-IR: Homeostasis model assessment index
HPLC: High-performance liquid chromatography
2hPPG: 2-hour postprandial blood glucose
HTC: Hepatic triglyceride content
IGT: Impaired glucose tolerance
JTW: Jiao Tai Wan
LDL-C: Low-density lipoprotein cholesterol
Met: Metformin
NAFLD: Nonalcoholic fatty liver disease
NEFA: Nonesterified fatty acid
OGTT: Oral glucose tolerance test
pACC: Phosphorylated-ACC
pAMPK: Phosphorylated-AMPK
SBP: Systolic blood pressure
STZ: Streptozotocin
TC: Total cholesterol
T2DM: Type 2 diabetes mellitus
TG: Triglycerides
WC: Waist circumference
WHR: Waist-to-hip ratio.
